# Simultaneous DNA and RNA isolation from brain punches for epigenetics

**DOI:** 10.1186/1756-0500-4-314

**Published:** 2011-08-30

**Authors:** Marc Bettscheider, Chris Murgatroyd, Dietmar Spengler

**Affiliations:** 1 Department of Molecular Neuroendocrinology, Max-Planck-Institute of Psychiatry, Kraepelinstr. 2-10, 80804 Munich, Germany

## Abstract

**Background:**

Epigenetic modifications such as DNA methylation play an important role for gene expression and are regulated by developmental and environmental signals. DNA methylation typically occurs in a highly tissue- and cell-specific manner. This raises a severe challenge when studying discrete, small regions of the brain where cellular heterogeneity is high and tissue quantity limited. Because gene expression and methylation are often tightly linked it appears of interest to compare both parameters in the same sample.

**Findings:**

We present a refined method for the simultaneous extraction of DNA for bisulfite sequencing and RNA for expression analysis from small mouse brain tissue punches. This method can also be easily adapted for other small tissues or cell populations.

**Conclusions:**

The method described herein results in DNA and RNA of a quantity and quality permitting highly reliable bisulfite analysis and quantitative RT-PCR measurements, respectively.

## Background

The spatio-temporal expression of a gene is defined by DNA sequence *(per se) *and the manner by which it is marked through epigenetic mechanisms including DNA methylation and chromatin modification. In eukaryotes DNA methylation typically comprises the covalent addition of a methyl group at the 5-position of cytosines that are followed by guanines, i.e. CpG dinucleotides. Functionally, DNA methylation frequently confers gene silencing.

CpG methylation of genomic DNA is routinely analyzed by the treatment of DNA with sodium bisulfite, followed by PCR amplification and sequencing [1]. While bisulfite readily deaminates cytosine residues to uracils, which are then converted to thymines during DNA amplification by PCR, 5-methylcytosine resists this modification. Many methods based on this principle have been developed including direct sequencing, pyrosequencing, methylation-specific PCR (MSP), combined bisulfite restriction analysis (COBRA), methylation-sensitive single nucleotide primer extension (MS-SNuPE) and microarray-based methods (for review see [2]).

Bisulfite analysis depends on high quantity and quality of DNA as the bisulfite conversion procedure itself requires long incubation times, elevated temperature, and high bisulfite concentration; all of which are highly detrimental to DNA [3]. Furthermore, to investigate the functional interrelationship between DNA methylation and RNA expression both should be determined within the same sample. In this respect, the analysis of expression data and DNA methylation from two separate cohorts of animals may introduce a bias, unless at least double the numbers of animals are included in each cohort. Similarly, the surgical splitting of tissues containing different cell types can confound the analysis as DNA methylation is highly tissue- and even cell-type specific. Finally, tissue punches of usually around 0.8 mm from distinct areas of the brain, are generally rather limiting.

Though a number of different methods have been developed for simultaneous extraction of DNA and RNA, a technique addressing efficient isolation from small tissue samples has not been reported so far. While TRIzol can be used for the simultaneous extraction of DNA and RNA, in addition to proteins [4], we note that that the quality of DNA produced from small tissues was not high enough for bisulfite analysis. Furthermore, we find that commercially available kits for RNA/DNA isolation, relying on spin-column purification [5], do not yield a high enough DNA quantity from small tissues to permit reliable bisulfite analysis (data not shown).

We have therefore adapted a derivative of the guanidinium thiocyanate-phenol-chloroform extraction method, originally devised by Piotr Chomczynski and Nicoletta Sacchi for the extraction of RNA [6]. While variants of a guanidinium thiocyanate-based (GTC) buffer have been used for RNA (for review see [7]), various forms of a guanidinium thiocyanate-based buffer have also proved efficient for the purification of DNA [8-12] and can be further modified for the simultaneous extraction of RNA and DNA in cancer tissues [13] and whole fish embryos [14]. Here we describe our experience in extracting both DNA and RNA from punched brain tissue and present an alternative for obtaining both DNA and RNA from the same cells for genome and transcriptome profiling. In addition, we characterized tissue specimens and cell quantities needed for this method.

## Materials and Methods

Tissue punches with 0.8 mm in diameter were taken from various brain regions including cortex, paraventricular nucleus (PVN) and dentate gyrus of C57/BL6 mice (Charles River) and were frozen at -80°C until nucleotide extraction. In addition smaller tissue punches of 0.3 mm diameter were taken from the cortex. To assess the sensitivity of the assay different numbers of Neuro2a (ATCC number CCL-131) cells were pelleted and subjected to the same isolation protocol. Initially we compared commonly used isolation methods to the simultaneous isolation of DNA and RNA from a single punch. Cortex punches (0,8 mm) were subjected to various DNA (Qiagen DNeasy Blood & Tissue Kit; CTAB method [15]; SDS/Proteinase K [16]; Gentra Puregene Tissue Kit) and RNA (Macherey Nagel NucleoSpin^® ^RNA II; TRIzol^® ^Reagent; Chomczynski protocol [7]) extraction protocols (Table 1).

**Table 1:** Yield and purity of DNA and RNA preparations from 0,8 mm cortex punches using commonly employed protocols, commercially available kits and the presented simultaneous DNA/RNA extraction method

Method DNA		Total yield [ng]	260/280	260/230
**(n = 6)**	Qiagen Dneasy	360 ± 96	1,8	1,35
	CTAB	520 ± 87	1,8	1,5
	SDS/Prot K	850 ± 195	2	1,4
	Gentra Puregene	360 ± 96	2	1,5
**Method RNA**				
**(n = 6)**	MN Nucleospin II	366 ± 75	2,1	0,2
	Trizol	690 ± 262	1,7	0,6
	Chomczynski	838 ± 321	1,9	1,4
**Simultaneous DNA/RNA**				
**(n = 9)**	DNA	765 ± 163	1,9	1,3
	RNA	330 ± 54	1,9	1,2

RNA/DNA yield, A260/280 and 260/230 ratios for the extracted samples were analyzed with an Implen Nanophotometer.

**Figure 1:**
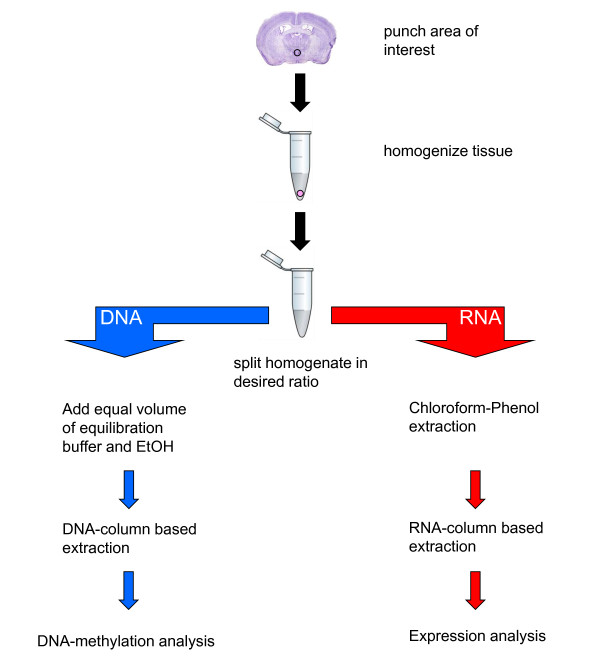
**Workflow for the simultaneous extraction of DNA and RNA**. The tissue punch is placed in 400 μl GTA buffer and vortexed until disrupted. The tissue is then completely homogenized by passing through a syringe (29G). The homogenate is then split for the purification of RNA and DNA. The split can be 1:1 or of different proportions depending on requirements of the experiment. The homogenate should be processed within a few hours.

For simultaneous extraction, tissue punches were homogenized using a pipette and vortexer in 400 μl of guanidinium thiocyanate (GTC) buffer (4.5 M guanidinium thiocyanate, 2% N-lauroylsarcosine, 50 mM EDTA pH 8, 25 mM Tris-HCl pH 7.5, 0.1 M beta-mercaptoethanol, 0.2% antifoam A) at room temperature and further passed several times through a hypodermic syringe (29G) (Figure 1). The guanidinium thiocyanate, along with the 2-mercaptoethanol and sarcosine, denatures proteins, including DNases and RNases. The lysate is then split into two equal parts and processed separately for RNA and DNA. Note that it is possible to divide the sample unequally depending on the demand for higher yields of either RNA or DNA. Given that RNA is far less stable than DNA, we suggest processing the RNA first while the DNA can be left for some hours at room temperature.

Briefly, for purification of RNA one half of the lysate is added to 1/10 volume of NaOAc, 1 volume acidic phenol and 1/2 volume of chloroform:isoamyl. The mixture is incubated on ice for 10 min, centrifuged for 20 min at 13,000 rpm at 4°C. The aqueous phase is transferred to a new tube and mixed with 70% EtOH to precipitate the RNA. The precipitate is then transferred to an RNA spin column (Macherey Nagel) and further processed according to the manufacturer's protocol including an on-column DNase-digestion. We found little difference in RNA quality or quantity between a number of commercially available spin columns (data not shown). Tissue punches as small as 0.8 mm diameter and 2,000 Neuro2a cells gave sufficient yield and quality of RNA (Table 2 and 3) to allow for RT-PCR analysis. However, yields of RNA from 0.3 mm punches were more variable suggesting that tissue quantity becomes a limiting factor.

**Table 2:** Yields of simultaneously isolated RNA and DNA from brain tissue punches (0,8 mm) of different brain regions and whole pituitary

Tissue	Total DNA yield [ng]	A260/280	260/230	Total RNA yield [ng]	A260/280	260/230
**PVN (n = 10)**	980	1,9	1,3	510	1,7	1

**DG (n = 39)**	1165	1,9	1,2	275	1,9	1,1

**Cortex (n = 9)**	675	1,9	1,4	330	1,9	1,2

**Pituitary (n = 29)**	11400	1,9	1,9	1425	1,78	1,8

**Table 3:** Yields of simultaneously isolated RNA and DNA from decreasing numbers of Neuro2a cells

Cell number (n = 3)	Total DNA yield [μg]	A260/280	A260/230	Total RNA yield [μg]	A260/280	A260/230
**500 000**	31 ± 1	2	2	12,7 ± 0,4	2	1,9

**200 000**	13 ± 0,8	2	2,1	5,4 ± 0,13	2	2

**100 000**	6,7 ± 0,6	2	2,1	2,8 ± 0,05	2	1,8

**50 000**	3,3 ± 0,25	2	1,4	1,44 ± 0,14	1,9	1,4

**20 000**	1,5 ± 0,07	1,9	1,5	0,59 ± 0,05	1,9	1,1

**10 000**	0,81 ± 0,07	2	1,1	0,41 ± 0,02	1,8	0,9

**5 000**	0,46 ± 0,06	1,9	0,9	0,29 ± 0,03	1,6	0,7

**2 000**	0,2 ± 0,05	1,9	0,6	0,25 ± 0,013	1,8	0,5

For purification of the DNA the other half of the lysate is equilibrated with equal volumes of Buffer AL and 100% EtOH and loaded on a Spin Column (Qiagen, DNeasy Blood and Tissue Kit). After subsequent washing steps according to the manufacturer's protocol the DNA is eluted with 70°C warm Buffer AE after a 10 min pre-incubation at 70°C. All tissue punches yielded enough DNA (Table 2) for sodium bisulfite treatment using 100 ng of DNA with the Epitect bisulfite kit (Qiagen). Starting material of less than 5,000 Neuro2a cells did not yielded satisfactory quantities of DNA (Table 3).

## Results and Discussion

DNA and RNA yields and absorbance rates for all methods studied are listed in Table 1. The most efficient method for brain tissue punches among the different DNA extraction protocols is the SDS/Proteinase K method with a total yield of 850 ng per punch followed by the CTAB method (550 ng) and the DNeasy and Puregene isolation Kits (both 360 ng). All methods resulted in A260/280 values between 1,8 and 2,0 indicating only minor protein contamination. For the tested RNA methods, the Chomczynski protocol yielded the highest amount of RNA per punch (838 ng) with an A260/280 ratio of 1,9, indicating rather pure RNA. The Trizol method resulted in slightly less RNA (690 ng) and low A260/280 values of 1,7 indicating some protein contamination. The MN Nucleospin columns gave the least amount of RNA (366 ng).

The newly developed simultaneous DNA and RNA extraction had the highest DNA recovery with 765 ng per punch considering that just half a punch is subjected to extraction. The RNA yield from the simultaneous extraction is comparable to the Trizol method, but with a higher purity as indicated by the A260/280 ratio of 1,9.

Low yields of DNA and RNA led to reduced 260/230 ratios for all methods tested, however, increasing the starting material improved this parameter (Table 3). Importantly, downstream applications such as PCR, RT-PCR, bisulfite treatment and subsequent bisulfite PCR are not compromised by lower 260/230 values, as shown below. Nevertheless, other downstream applications should be tested prior to sample processing.

**Figure 2:**
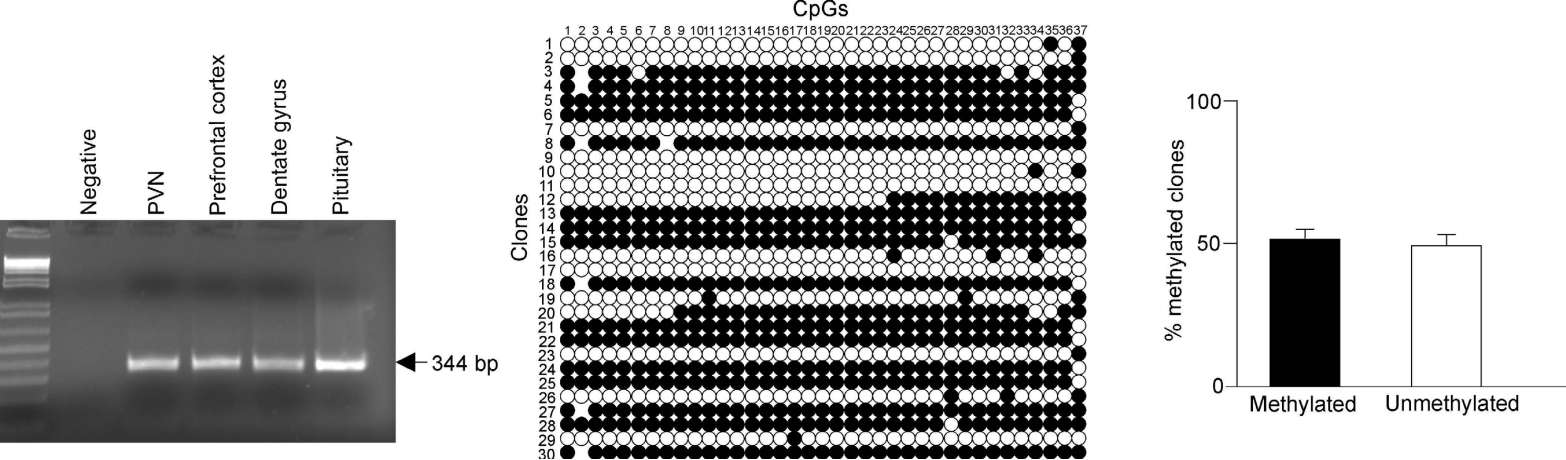
**Bisulfite sequencing of DNA from brain tissue punches**. DNA (100 ng) from PVN tissue punches (n = 5) were separately bisulfite treated. Bisulfite primers directed against the mouse *Zac1 *differentially methylated region (DMR) were used in a PCR. The amplified products were then cloned and DNA from 30 separate recombinant colonies sequenced. Data analysis revealed that the *Zac1 *locus is methylated at levels of 52% ± 5% corresponding to current literature that the gene is maternally imprinted.

The suitability of the extracted DNA for bisulfite analysis was investigated by using primers (F, ggtattaggtttagagtttatt; R, ttctccaacctcactcrccta) corresponding to the imprinted *Zac1 *gene [17]. The promoter of the maternally imprinted *Zac1 *gene is DNA methylated on only the maternal derived allele; therefore bisulfite sequencing should yield ratios of 50% methylation at each CpG in the promoter if the extracted DNA is of a sufficient quality and quantity. Indeed, we detected that DNA from PVN tissues gave an average of 52% ± 5% (Figure 2) supporting that DNA isolated from relatively small brain punches allow for robust DNA methylation analysis.

**Figure 3:**
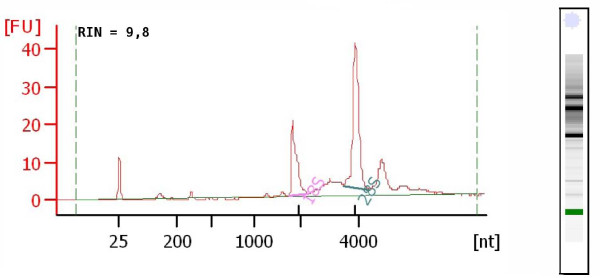
**Quality of RNA extracted from brain tissue punches**. Representative Bioanalyzer 2100 electropherogram of extracted RNA. Quality and integrity of the RNA was measured using the Agilent Bioanalyzer 2100 yielding RIN scores ranging from 8-10.

The quality of the extracted RNA was further examined using the Agilent Bioanalyzer 2100 resulting in RIN scores ranging from 8-10 (Figure 3) indicating undegraded RNA of high quality. The RNA could be further reverse transcribed (Superscript II, Invitrogen) and produced reliable products with a number of housekeeping and tissue specific genes.

**Figure 4:**
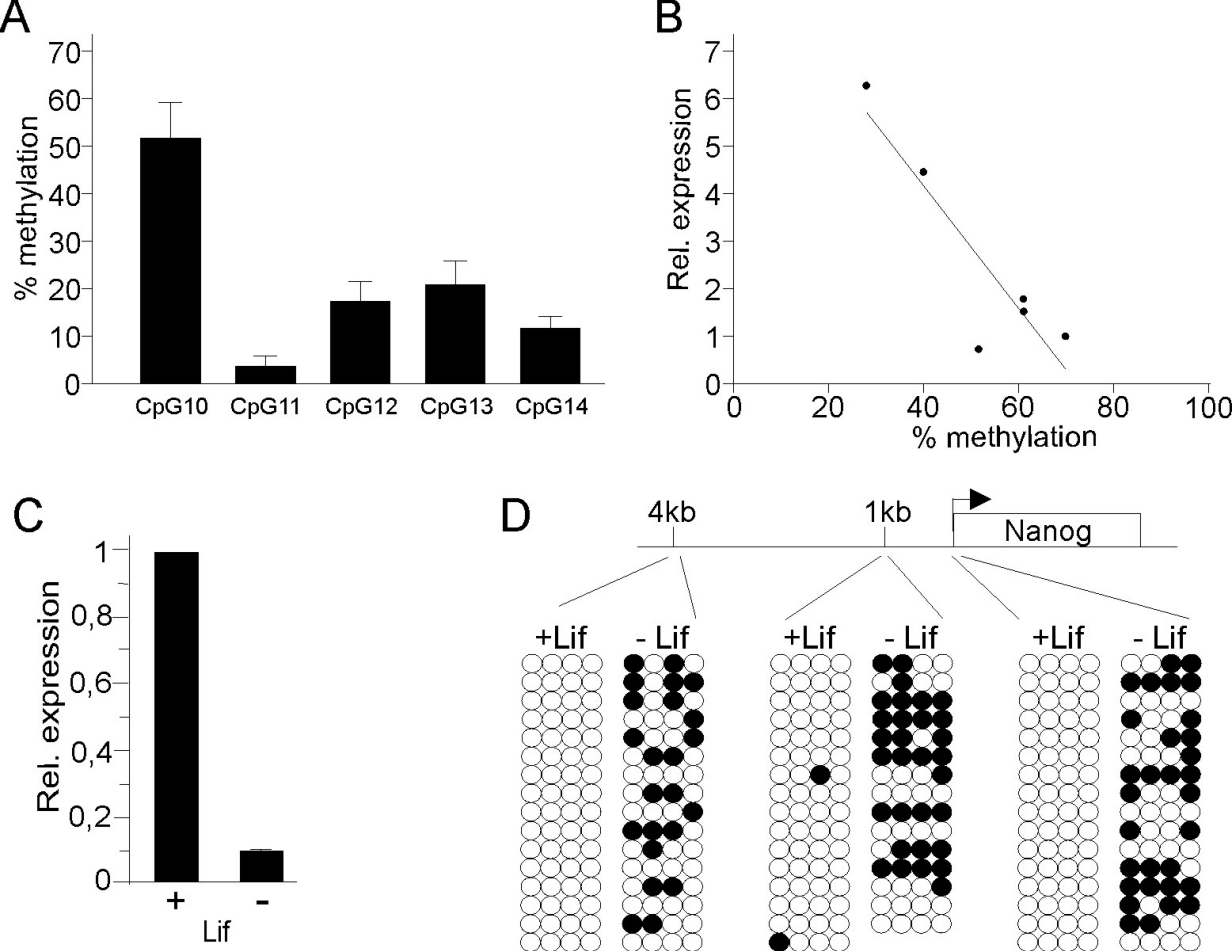
**Analysis of gene transcription and methylation from stem cells and brain punches**. (A) DNA and RNA were simultaneously extracted from PVN tissue punches of Bl6 mice. Bisulfite treated (Qiagen) DNA was amplified using bisulfite primers directed against a region of the mouse AVP gene enhancer [18]. The amplified products were then cloned in a pGEM vector (Promega) and 16 recombinant colonies sequenced using T7 primer. (B) RNA (100 ng) was reverse transcribed and then subjected to quantitative realtime PCR using primers against AVP cDNA [18]. *AVP *gene expression was correlated with methylation at individual CpGs revealing that methylation at CpG10 DNA negatively correlates with *AVP *expression (R^2 ^= 0.8259). (C) Differentiated [+Lif] and undifferentiated [-Lif] mouse ES cells [19] were simultaneously extracted for RNA and DNA. RNA (100 ng) was reverse transcribed and then subjected to quantitative realtime PCR (Nanog F - agggtctgctactgagatgctctg, R - caaccactggtttttctgccaccg) and normalized to the expression of the housekeeping gene *HPRT *(F - acctctcgaagtgttggatacagg, R - cttgcgctcatcttaggctttg). (D) DNA (100 ng) from undifferentiated and differentiated cells was bisulfite treated and amplified using primers directed against the indicated regions of the mouse *Nanog *gene (4kb F - tttgtaggtgggattaattgtgaat, R - aaaaaaacaaaacaccaaccaaat; 1Kb F - ggaagattaggagtttgggattagt, R - atctaccaccatacccaatttaaaa; proximal promoter F - ggtagtttgttgggttttgtatttt, R - aactcttatctccccattcctaaac) and 16 recombinant colonies sequenced. Following differentiation the *Nanog *promotor becomes methylated and gene expression silenced.

The DNA and RNA extracted from this procedure was also tested for simultaneous bisulfite sequencing and quantitative real time RT-PCR experiments. As a proof of principle we investigated 7 PVN samples for *Arginine Vasopressin *(*AVP*) expression and DNA methylation and confirmed negative correlations (Figure 4A,B) as previously described [18]. Moreover, using this method we have been able to study DNA methylation and gene expression during differentiation of embryonic stem cells. We find that differentiation of mouse ES cells [19] leads to silencing of the pluripotency marker gene, *Nanog*, and this correlates with methylation of promoter regions, important in *Nanog *regulation [20] (Figure 4C,D).

In summary, we have refined and verified a streamlined protocol for the isolation of high qualities and quantities of DNA and RNA from small tissues for the study of DNA methylation and mRNA expression. Such a procedure will facilitate the analysis of the role of DNA methylation on gene expression through direct correlation analysis.

## Competing interests

The authors declare that they have no competing interests.

## Authors' contributions

MB and CM performed the experiments. All authors contributed to the conception, design, analysis, interpretation of the data and drafting of the manuscript.
